# Two-color STED microscopy reveals different degrees of colocalization between hexokinase-I and the three human VDAC isoforms

**DOI:** 10.1186/1757-5036-3-4

**Published:** 2010-03-05

**Authors:** Daniel Neumann, Johanna Bückers, Lars Kastrup, Stefan W Hell, Stefan Jakobs

**Affiliations:** 1 Max Planck Institute for Biophysical Chemistry, Department of NanoBiophotonics, Am Fassberg 11, 37077 Göttingen, Germany

## Abstract

The voltage-dependent anion channel (VDAC, also known as mitochondrial porin) is the major transport channel mediating the transport of metabolites, including ATP, across the mitochondrial outer membrane. Biochemical data demonstrate the binding of the cytosolic protein hexokinase-I to VDAC, facilitating the direct access of hexokinase-I to the transported ATP. In human cells, three hVDAC isoforms have been identified. However, little is known on the distribution of these isoforms within the outer membrane of mitochondria and to what extent they colocalize with hexokinase-I. In this study we show that whereas hVDAC1 and hVDAC2 are localized predominantly within the same distinct domains in the outer membrane, hVDAC3 is mostly uniformly distributed over the surface of the mitochondrion. We used two-color stimulated emission depletion (STED) microscopy enabling a lateral resolution of ~40 nm to determine the detailed sub-mitochondrial distribution of the three hVDAC isoforms and hexokinase-I. Individual hVDAC and hexokinase-I clusters could thus be resolved which were concealed in the confocal images. Quantitative colocalization analysis of two-color STED images demonstrates that within the attained resolution, hexokinase-I and hVDAC3 exhibit a higher degree of colocalization than hexokinase-I with either hVDAC1 or hVDAC2. Furthermore, a substantial fraction of the mitochondria-bound hexokinase-I pool does not colocalize with any of the three hVDAC isoforms, suggesting a more complex interplay of these proteins than previously anticipated. This study demonstrates that two-color STED microscopy in conjunction with quantitative colocalization analysis is a powerful tool to study the complex distribution of membrane proteins in organelles such as mitochondria.

**PACS:** 87.16.Tb, 87.85.Rs

## 1. Introduction

The voltage-dependent anion-selective channels are the most abundant proteins in the outer membrane of mitochondria [1,2]. VDACs are small (30-35 kDa) pore-forming proteins that are ubiquitous to all eukaryotes [3]. They are the major channels for the passage of ions and small molecules, including NADH and ATP across the mitochondrial outer membrane [4]. The regulation of the transport rates of these metabolites has been suggested to influence organellar and cellular metabolism, setting VDAC at a central position in the regulation of cellular energy metabolism. In humans, three different isoforms (hVDAC1, hVDAC2, hVDAC3) exist. They can be found in most tissues, albeit at different amounts [5,6]. VDAC exhibits numerous interactions with mitochondrial and cytosolic proteins [7,8] and even with components of the cytoskeleton [9,10]. Furthermore, VDAC has been reported to bind to pro- and anti-apoptotic proteins of the Bcl-2 family and has been proposed to be a major player in mitochondria mediated apoptosis, although its precise role is controversially discussed [11-15].

A well-studied interaction is the binding of VDAC to the cytosolic protein hexokinase-I [16,17]. Hexokinase-I is highly expressed in brain, but is also prevalent in other tissues [16,18]. The binding of hexokinase-I to VDAC facilitates its access to ATP and it has been suggested that hexokinase-I modulates VDACs role in apoptosis [19,20].

Notably, early studies on VDAC characterized this protein as the outer membrane hexokinase binding factor [21]. VDAC may enhance binding of hexokinase-I, but it is not essential for the binding of hexokinase-I to the outer membrane. Necessary and sufficient for mitochondrial binding of hexokinase-I is a 15 amino acid long N-terminal domain that inserts into the outer membrane [22].

Biochemical data indicates that only VDAC1 but not VDAC2 binds hexokinase [23], although this finding was questioned by a different study [24]. Hence, currently, little quantitative data is available on the extent to which hexokinase is associated with the various VDAC isoforms and vice versa. Likewise, little is known on potential differences in the sub-mitochondrial distribution of the three VDAC isoforms and mitochondrial associated hexokinase [25].

The detailed sub-mitochondrial distributions of VDAC and hexokinase are difficult or even impossible to address using conventional light microscopy, because many proteins in the mitochondrial outer membrane are too densely packed to be resolved [26,27]. The challenge is that the resolution of conventional lens-based (far-field) fluorescence microscopy is limited by diffraction to about a half of the wavelength of light (*λ*/2~200 nm) within the optical plane and to about *λ *(~500 nm) along the optical axis.

Recently, however, several lens-based (far-field) fluorescence microscopy approaches emerged that are no longer constrained in resolution by the wavelength of light [28,29]. The first concrete and viable concept was stimulated emission depletion (STED) microscopy, where the fluorophores located at the outer rim of a scanning focal spot of excitation light are transiently switched off by de-excitation through stimulated emission [30,31]. As a consequence, only fluorophores within an effective focus with a diameter of *d *≈ *λ*/(2*n *sin *a *) are able to fluoresce [32]. *I*_*s *_is a characteristic of the fluorophore, whereas *I *is the intensity of the STED-beam inducing the de-excitation. For *I/I*_*s *_→ ∞ it follows that d → 0. Hence, unlike in conventional lens-based optical microscopes, the resolution is no longer fundamentally limited by the wavelength.

Two-color STED microscopy has previously been used to study protein distributions within mitochondria [27,33] and other cellular compartments [34]. However, STED images have so far not been utilized for rigorous colocalization analysis to obtain insights into the relative distributions of interacting proteins. In this study we show that hVDAC1 and hVDAC2 are distributed into the same distinct domains in the outer membrane, whereas hVDAC3 is mostly uniformly distributed over the surface of the mitochondrion. STED microscopy demonstrates that hexokinase-I and hVDAC3 are both located in distinct clusters in the outer membrane, which are not resolvable by conventional microscopy. We quantitatively analyze two-color STED microscopy images to determine the level of colocalization between hexokinase-I and the three hVDAC isoforms.

## 2. Methods

### 2.1 Sequence alignment

hVDAC1 (NP_003365), hVDAC2 (NP_003366) and hVDAC3 (NP_005653) sequences were taken from the NCBI database. The multiple sequence alignment was performed with the ClustalW2 program [35] from the EMBL-EBI homepage using the default values http://www.ebi.ac.uk/Tools/clustalw2/index.html.

### 2.2 Cloning

Expression plasmids for the following fusion constructs were generated: hVDAC1-Flag, hVDAC1-V5, hVDAC2-Flag and hVDAC3-Flag. The expression plasmids pDest40-hVDAC1-V5 and pDestMD-hVDAC2-Flag were created by gateway cloning (Invitrogen, Carlsbad, CA) using DONR clones from the Human ORFeome Collection [36] and V5 or Flag containing DEST vectors, respectively. For the generation of the hVDAC1-Flag and hVDAC3-Flag expression vectors, the coding sequences of hVDAC1 and hVDAC3 were amplified by PCR using the following primers: ATC GGT ACC AAT GGC TGT GCC ACC CAC G and CCG GGA TCC TGC TTG AAA TTC CAG TCC T for hVDAC1 and ATC GGT ACC AAT GTG TAA CAC ACC AAC GT and CCG GGA TCC AGC TTC CAG TTC AAA TCC C for hVDAC3. Subsequently the PCR products were digested and inserted into the *KpnI *and *BamHI *restriction sites of the multiple cloning site of pFlag-CMV-5.1 (Sigma-Aldrich, Taufkirchen, Germany).

### 2.3 Cell culture and transfection

Human osteosarcoma (U2OS) cells (ECACC, Salisbury, UK) were cultivated in DMEM medium (Invitrogen, Carlsbad, CA) with Glutamax and 4.5% (w/v) Glucose supplemented with 50 U/ml Penicillin, 50 *μ*g/ml (w/v) Streptomycin, 1 mM Na-Pyruvate and 10% (v/v) FCS (Invitrogen, Carlsbad, CA). The cells were cultured in petri dishes at 37°C and 7% CO_2_. For transfection, the Optifect reagent (Invitrogen, Carlsbad, CA) was used according to the manufacturer's instructions. The cells were imaged 24 hours after the transfection.

### 2.4 Sample preparation

For immunolabeling, cells were grown on coverslips over night, fixed with 8% (w/v) formaldehyde in phosphate buffered saline (PBS) (137 mM NaCl, 3 mM KCl, 8 mM Na_2_HPO_4_, 1.5 mM KH_2_PO_4_, pH 7) for 5 minutes at 37°C, extracted for 5 min with 0.5% (v/v) Triton X-100 in PBS, blocked with PBS with 10% bovine serum albumin (BSA) for 5 min and incubated with the following primary antibodies in PBS with 10% BSA for 1 hour: anti-Tom20 rabbit polyclonal antibody (Santa Cruz Biotechnology, Santa Cruz, CA), anti-hexokinase-I rabbit monoclonal antibody (Cell Signaling Technology, Danvers, MA), anti-Flag monoclonal mouse antibody (Sigma-Aldrich, Taufkirchen, Germany), or anti-V5 polyclonal rabbit antibody (Sigma-Aldrich, Taufkirchen, Germany). Then, after 5 minutes incubation with 0.5% (v/v) Triton X-100 in PBS, followed by 5 minutes blocking with PBS with 10% BSA, the coverslips were incubated with the following secondary antibodies: goat anti-rabbit antibody conjugated to Alexa 488 (Invitrogen, Carlsbad, CA), sheep anti-mouse antibody conjugated to Cy 3 (Dianova, Hamburg, Germany). For STED microscopy, sheep anti-mouse, goat anti-rabbit and donkey anti-goat antibodies (Jackson ImmunoResearch Laboratories, West Grove, PA) custom-labelled with ATTO 590 (ATTO-TEC GmbH, Siegen, Germany) or KK114 (kindly provided by Dr. K. Kolmakov and Dr. V. Belov, Dept. of NanoBiophotonics, Max Planck Institute for Biophysical Chemistry) were used. Finally, the samples were washed in PBS for 30 minutes and mounted in Mowiol (Sigma-Aldrich, Taufkirchen, Germany) with 0.1% (w/v) DABCO (Sigma-Aldrich, Taufkirchen, Germany) and, occasionally, with DAPI (Sigma-Aldrich, Taufkirchen, Germany).

### 2.5 Microscopy

Confocal images were recorded with a beam scanning confocal microscope (TCS SP5, Leica Microsystems) equipped with an HCX PL APO 63×/1.40-0.60 oil objective lens. For three color image acquisitions the microscope was used in the sequential mode. The fluorescence was excited at 405 nm (DAPI), 488 nm (Alexa 488) and 561 nm (Cy 3). The detection wavelength range was set to 410-435 nm for DAPI, 495-520 nm for Alexa 488 and 570-650 nm for Cy 3. Pixel dimensions were chosen according to the Nyquist criterion. Each image was averaged twice. Except for contrast stretching, no other image processing was applied.

Two-color STED images were recorded with a custom-built STED-microscope which combines two pairs of excitation and STED laser beams all derived from a single supercontinuum laser source similar to the one described previously [37]. The excitation wavelengths (570 ± 5 nm for ATTO 590, 650 ± 5 nm for KK 114) were selected from the supercontinuum using an acousto-optical tunable filter (AOTF, AA Opto-Electronic, Orsay Cedex, France) whereas the STED wavelengths (710 ± 20 nm for ATTO 590, 755 ± 20 nm for KK 114) were extracted using prism monochromators and appropriate interference bandpass filters. All beams were coupled into separate polarization maintaining single-mode fibers. At the fiber outputs the beams were collimated and sent to the objective lens. Doughnut-shaped STED foci with a zero-intensity minimum in the center were generated by placing two vortex phase plates (RPC Photonics, Rochester, NY, U.S.A.) into the STED beams. The excitation and STED beams were combined into pairs of beams with orthogonal polarization using dichroic beamsplitters. The two pairs of excitation and STED beams were then combined with a polarizing beam splitter cube and coupled into the objective lens. An achromatic quarterwave plate (500-900 nm, B. Halle GmbH, Berlin, Germany) placed at the back of the objective lens afforded circular polarization for all beams. The fluorescence from both fluorophores was first separated from the laser beams with a custom multi-band dichroic beamsplitter (Chroma Technology, Rockingham, VT, USA), was split up into the two colors with a second dichroic beamsplitter (Z635RDC, Chroma Technology) and was further cleaned up with bandpass and longpass filters (620/40 m for ATTO 590 and 670/40 m + Z660LP for KK 114, all from Chroma Technology). The fluorescence was focused into separate multimode optical fibers (M31L01, Thorlabs, Dachau/Munich, Germany) which served as confocal pinholes. The fibers were attached to single-photon counting modules (SPC-AQRH-13-FC, Perkin Elmer, Salem, MA, USA) which were in turn connected to custom time gating electronics which separated the fluorescence signals following each of the excitation/STED pulse pairs.

The spatial alignment of the excitation and the STED foci was made by sequential measurement of the point spread functions (PSFs) of the four beams. The PSFs were acquired by recording the scattered light from gold nanoparticles (80 nm) as they were scanned through the focus. The alignment precision was better than 20 nm, i.e. below the resolution achievable with the setup and, consequently, beam offsets could be neglected for the analysis of the recorded images.

To ensure the simultaneous arrival of the excitation and the respective STED beams in the sample, the optical path lengths were matched for each pair of beams. However, the pairs of excitation and STED beams for the two different color channels were time-shifted by about 40 ns. This pulse-interleaved acquisition scheme allowed the (quasi-)simultaneous recording of both color channels while the fluorescence from the two color channels could be separated based on the photon arrival time at the detector. Therefore, no shift of the images is expected, e.g. due to a drift of the sample, which would compromise the analysis of the data.

Typically, the images were acquired with pixel size of 20 × 20 nm^2 ^and a pixel dwell-time of 1 ms. For comparison, confocal images were recorded as a reference in addition to the STED images. All image data shown in this study and used for subsequent analysis are raw data. No deconvolution algorithms were utilized.

### 2.6 Image analysis

**Figure 1:**
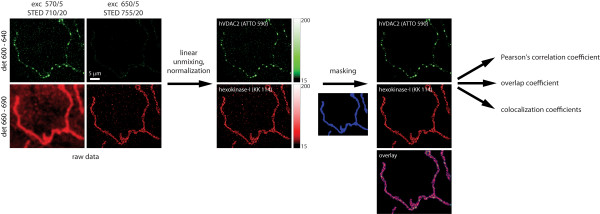
**Image analysis**. The two-color STED images were collected with an interleaved pulse scheme. The first pair of excitation/STED pulses (at wavelengths of 650 and 755 nm) was followed by the second pair (at wavelengths of 570 and 710 nm) with a 40 ns delay. Using two detectors and a time-gated detection scheme, it was thus possible to detect the fluorescence emission following each pulse in two different wavelength bands, resulting in a set of four recordings. The four raw images were linearly unmixed to correct for channel crosstalk and were normalized. Segmentation was then performed with an iterative seeded region growing algorithm which extracted the regions of the image containing mitochondria. Only those regions were used in the subsequent calculation of the correlation coefficients. For better visibility the images are shown with a logarithmic color map.

The image analysis was performed with custom routines written in MATLAB (The Mathworks, Natick, MA, USA). Before analyzing the colocalization of each pair of proteins, the raw data were pre-processed to correct for imaging artifacts which interfere with the colocalization analysis. In particular, crosstalk between the two detection channels was removed and masks were applied. The workflow of the image analysis is shown in Fig. 1.

Due to the spectral overlap between the absorption and emission spectra of the dyes ATTO 590 and KK 114, their fluorescence emission cannot be strictly separated by wavelength-selective fluorescence excitation and/or detection. Rather, the emission of the ATTO 590 dye is not only recorded in the short wavelength ("green") detection channel but, to some degree, also in the long wavelength ("red") channel. The same holds for the KK 114 emission which, to a minor extent, is also registered by the "green" channel. However, the employed image acquisition scheme minimizes the crosstalk by using both spectrally selective excitation and detection. The remaining cross talk was eliminated by means of linear unmixing [38] where the crosstalk matrix had been previously determined from singly labeled reference samples.

After normalization, the images were masked to restrict the colocalization analysis to the regions containing mitochondria. To compute the mask, a seeded region growing algorithm was employed. Initially, the measured images were eroded with a disk (*r *= 3-5 pixels) to remove small isolated spots of fluorescence which were present in some of the images and were probably due to unspecifically bound single antibodies. Next, the images were slightly smoothed with a Gaussian filter (*σ *= 3-5 pixels) and were normalized. Seed points were then generated by thresholding the smoothed image at a level of (*μ *+ *xσ*) where *μ *is the mean image intensity, *σ *is its standard deviation and *x *is a signal-to-noise paramater which was chosen between 0.8 (confocal images) and 1.8 (STED images). Starting from these seed points, region growing (implemented as repeated dilation/thresholding operations) was applied until the pixel intensity dropped below *t*_a _times the average signal intensity (as determined from the masked regions) or reached *t*_b _times the average background intensity (as determined from the unmasked regions); *t*_a _was typically chosen between 0.3 and 0.75 whereas *t*_b _= 3.4. The masks were generated for both color channels individually and were combined with a logical OR operation to get the final mask.

For the actual colocalization analysis we decided to employ several colocalization measures each of which have their individual strengths and limitations [39,40]. One common choice to quantify the colocalization of two features is Pearson's correlation coefficient [41]

where the summations extend over all pixels, *R*_*i *_and *G*_*i *_are the intensity values of pixel *i *in the "red" and "green" channels and  and  are their mean values, respectively. Because the mean intensities are subtracted from the pixel values, *r*_p _can take values between -1 and +1. For the same reason, overlap between randomly located features does not increase the correlation coefficient but averages out to zero. Because negative values of *r*_p _are difficult to interpret and *r*_p _depends on the relative number of features present in the two channels, we additionally adopted other measures of colocalization in our analysis. The overlap coefficient [39]

measures the fraction of all ("red" and "green") objects which colocalize in the two images and therefore ranges from 0 to 1. It is more intuitively interpreted than Pearson's correlation coefficient but its value also depends on the relative number of features present in the two channels. Further, its significance is compromised by randomly distributed and overlapping features which do not cancel out as is the case for *r*_p_. To account for different numbers of features in the two channels, the colocalization coefficients have been introduced [39]:

where *R*_*i*, coloc _= *R*_*i *_if *G*_*i *_> 0 and *G*_*i*, coloc _= *G*_*i *_if *R*_*i *_> 0. *M*_1 _and *M*_2 _represent the fraction of features in one channel which colocalize with a feature in the other channel and are thus a direct and easily interpretable measure to quantify colocalization. Their reliability, however, is compromised by the requirement to set thresholds to classify pixels as objects or background. Our analysis showed that the values of *M*_1 _and *M*_2 _critically depend on the choice of the thresholds.

## 3. Results and discussion

### 3.1 hVDAC1 and hVDAC2 exhibit different distributions within the mitochondrial outer membrane than hVDAC3

**Figure 2:**
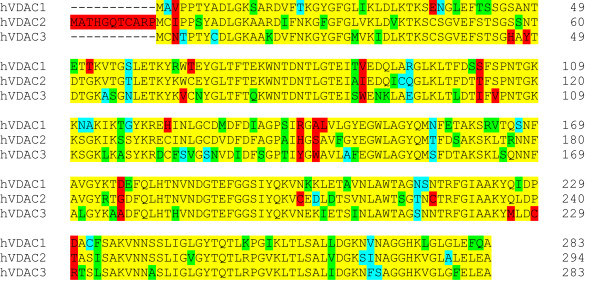
**Amino acid sequence alignment of the three hVDAC isoforms**. Identical amino acids are colored in yellow, conserved substitutions in green and semi-conserved substitutions in turquoise. Substitutions with no similarity are red colored. hVDAC1 and hVDAC2 exhibit 72% identity and 91% similarity, hVDAC2 and hVDAC3 share 71% identical and 91% similar amino acids and hVDAC1 and hVDAC3 have 67% identical and 94% similar amino acids.

The hVDAC1, hVDAC2, and hVDAC3 genes encode proteins with identities ranging between 67% and 72%. The majority of the non-identical amino acid residues is replaced by conserved or semi-conserved substitutions, thus the three proteins are highly homologous (similarities > 90%) on the amino acid level (Fig. 2).

In order to investigate the distribution of the three hVDAC isoforms within mitochondria, we genetically fused the coding sequence of the eight amino acid long Flag tag or of the 14 amino acid long V5 tag to the respective cDNAs. The fusion constructs were expressed in U2OS cells, a human osteosarcoma cell line, and were subsequently immunolabeled with specific antisera against either the Flag or the V5 tag and imaged with a confocal scanning microscope.

**Figure 3:**
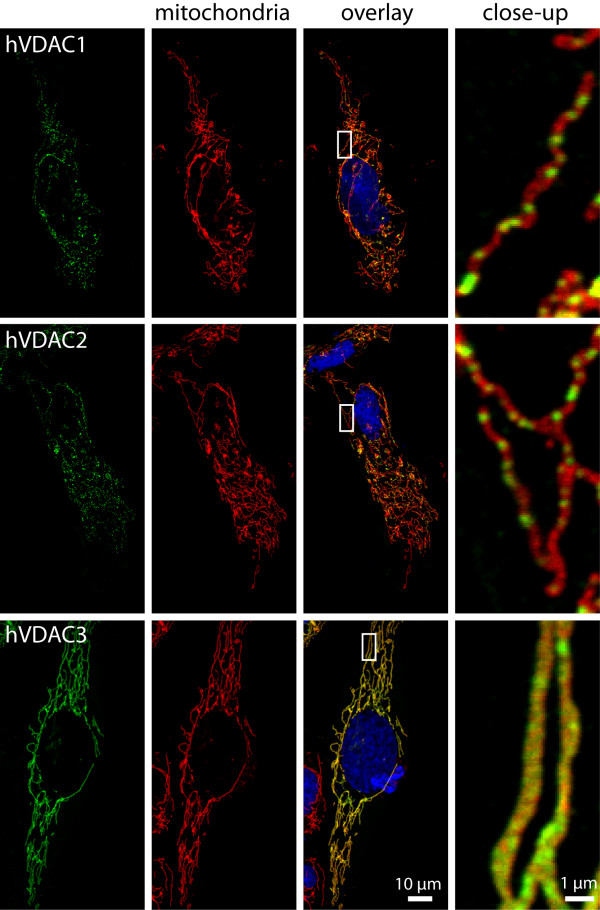
**Distinct sub-mitochondrial distributions of the three hVDAC isoforms**. Confocal microscopy of immunolabeled cultured human U2OS cells. Green: Different hVDAC isoforms, as indicated. For visualization, the respective Flag-tag fusion proteins were decorated with an antiserum against the Flag-peptide. Red: Mitochondria, highlighted with antibodies against the mitochondrial protein Tom20. Blue: Nucleus, stained with DAPI. The close-ups of the overlays show that hVDAC1 and hVDAC2 are localized in distinct domains, whereas hVDAC3 is uniformly distributed along the mitochondrial tubules.

In U2OS cells, as in most cultivated mammalian cells, the mitochondria assume a tubular shape and are connected to highly dynamic loose networks (Fig. 3). The three hVDAC isoforms localize exclusively to the mitochondria, as demonstrated by the colocalization with the mitochondrial marker Tom20 (Fig. 3). Using confocal scanning microscopy we found that hVDAC1 and hVDAC2 localized in most cells in relatively large domains (diameter ~300-900 nm) that were distributed along the mitochondrial tubules. To our knowledge, no other mitochondrial protein has been described to localize in comparable large domains in the outer membrane. In contrast to hVDAC1 and hVDAC2, hVDAC3 was largely homogenously distributed along the length of the mitochondrial tubule (Fig. 3). Interestingly, phylogenetic analysis indicates that VDAC3 is the the more primordial of the vertebrate VDAC genes and that the more closely related VDAC1 and VDAC2 arised more recently [3,42]. One might speculate that the different localizations could reflect different cellular functions of hVDAC1 and hVDAC2 in comparison to hVDAC3, possibly related to apoptosis, as only VDAC1 and VDAC2 have been discussed to play a role in this process [11,12,43-45]. Clearly, this suggestion requires further study.

### 3.2 hVDAC1 and hVDAC2 colocalize in the same domains

**Figure 4:**
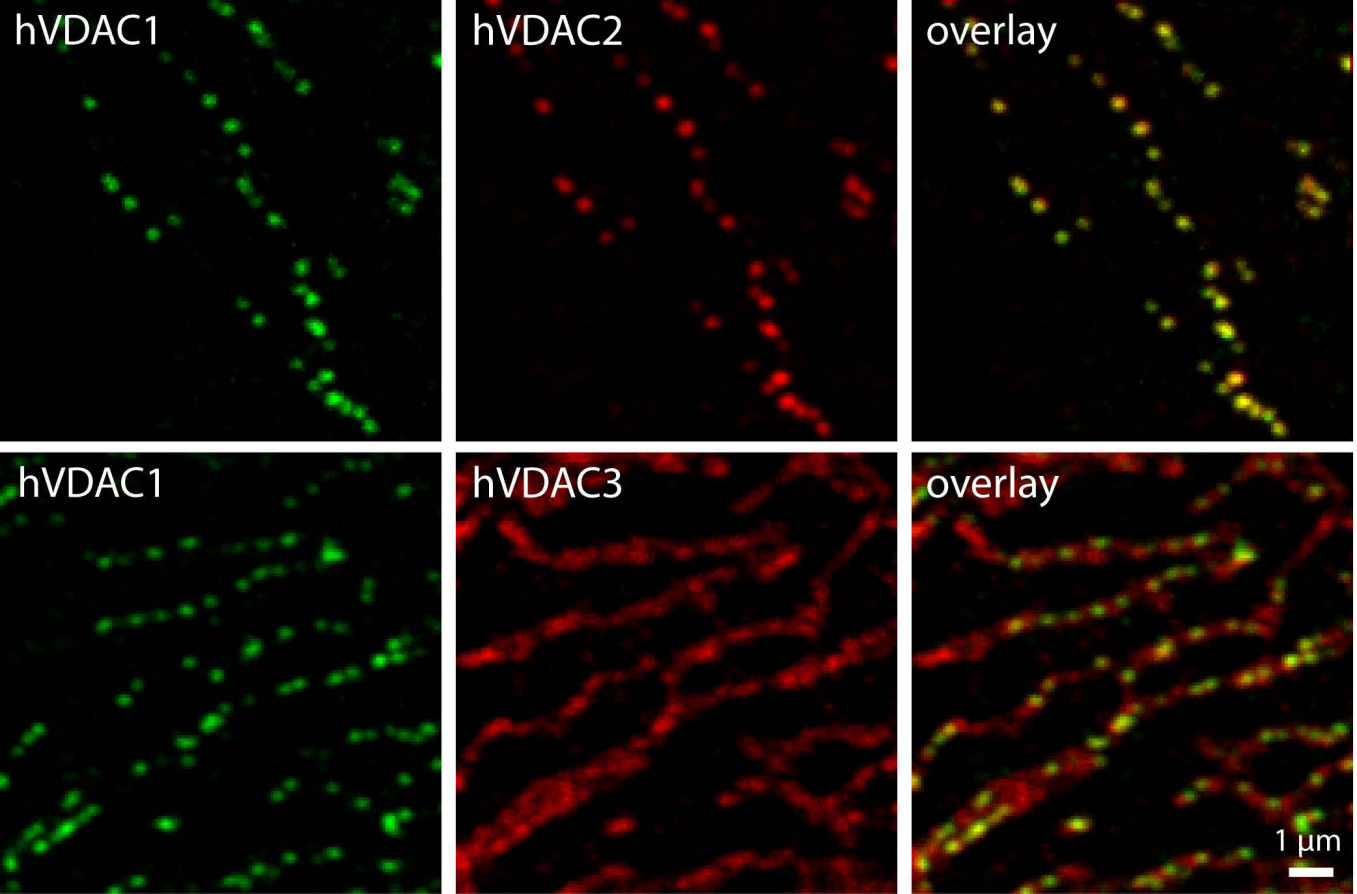
**hVDAC1 and hVDAC2 colocalize in distinct domains in the mitochondrial outer membrane**. Confocal imaging of cells expressing two tagged hVDAC isoforms. U2OS cells were decorated with antibodies against the Flag and the V5 tag. The fluorescence signals of hVDAC1-V5 and hVDAC2-Flag overlap to a large extent in confined domains, whereas hVDAC3-Flag has a distinct distribution. For a quantitative analysis of the colocalization see Additional file 3.

This pronounced difference in the sub-mitochondrial localizations was corroborated when the different VDAC isoforms were co-expressed (Fig. 4). We found an apparently entire overlap of the hVDAC1 and hVDAC2 signals, suggesting a complete colocalization of these two proteins. In all experiments, the distribution of hVDAC3 was distinctly uniform as compared to the other two isoforms. However, we found that upon co-expression of hVDAC3 with hVDAC1 or with hVDAC2, some heterogeneity in the VDAC3 distribution was induced (Fig. 4), which was not observed when hVDAC3 was expressed alone (for comparison see Additional file 1). These frequently hardly discernible hVDAC3 clusters were spatially overlapping with hVDAC1 or hVDAC2. We experimentally excluded bleedthrough of the respective fluorescence signals, (Additional file 2), thus this observation suggests an interaction between hVDAC1 or hVDAC2 with hVDAC3, which induces a slight but recognizable change in the distribution of hVDAC3. Still, also in cells co-expressing tagged hVDAC3 with one of the other isoforms, we find hVDAC3 mostly distributed along the mitochondrial surface and hence only partially colocalizing with hVDAC1 or hVDAC2. This visual impression is fully corroborated by a statistical analysis on a larger data set (Additional file 3).

We conclude that although the three hVDAC isoforms exhibit a very high degree of similarity in their primary structure, they have different outer membrane distributions. hVDAC1 and hVDAC2 colocalize in the same relatively large domains, whereas hVDAC3 is uniformly distributed in the mitochondrial outer membrane.

### 3.3 Two-color STED microscopy reveals individual hVDAC3 and hexokinase-I clusters

The interaction between hVDAC1 and hexokinase-I is biochemically well described, whereas the interaction between hVDAC2 and hexokinase-I is controversially discussed [23,24]. To the best of our knowledge, no data is available on the direct interaction between hVDAC3 and hexokinase-I. Hexokinase has been reported to localize to mitochondria from brain and liver as well as to the mitochondria of cancer cells, including the U2OS cells utilized in this study [22,46]. In principle, colocalization analysis based on fluorescence micrographs could give new insights into the degree of direct interaction between the various hVDAC isoforms and hexokinase-I. However, the fact that hVDAC3 (Fig. 3) appeared to be homogenously distributed in the mitochondrial outer membrane when imaged with conventional microscopy, immediately foreclosed a meaningful colocalization analysis.

**Figure 5:**
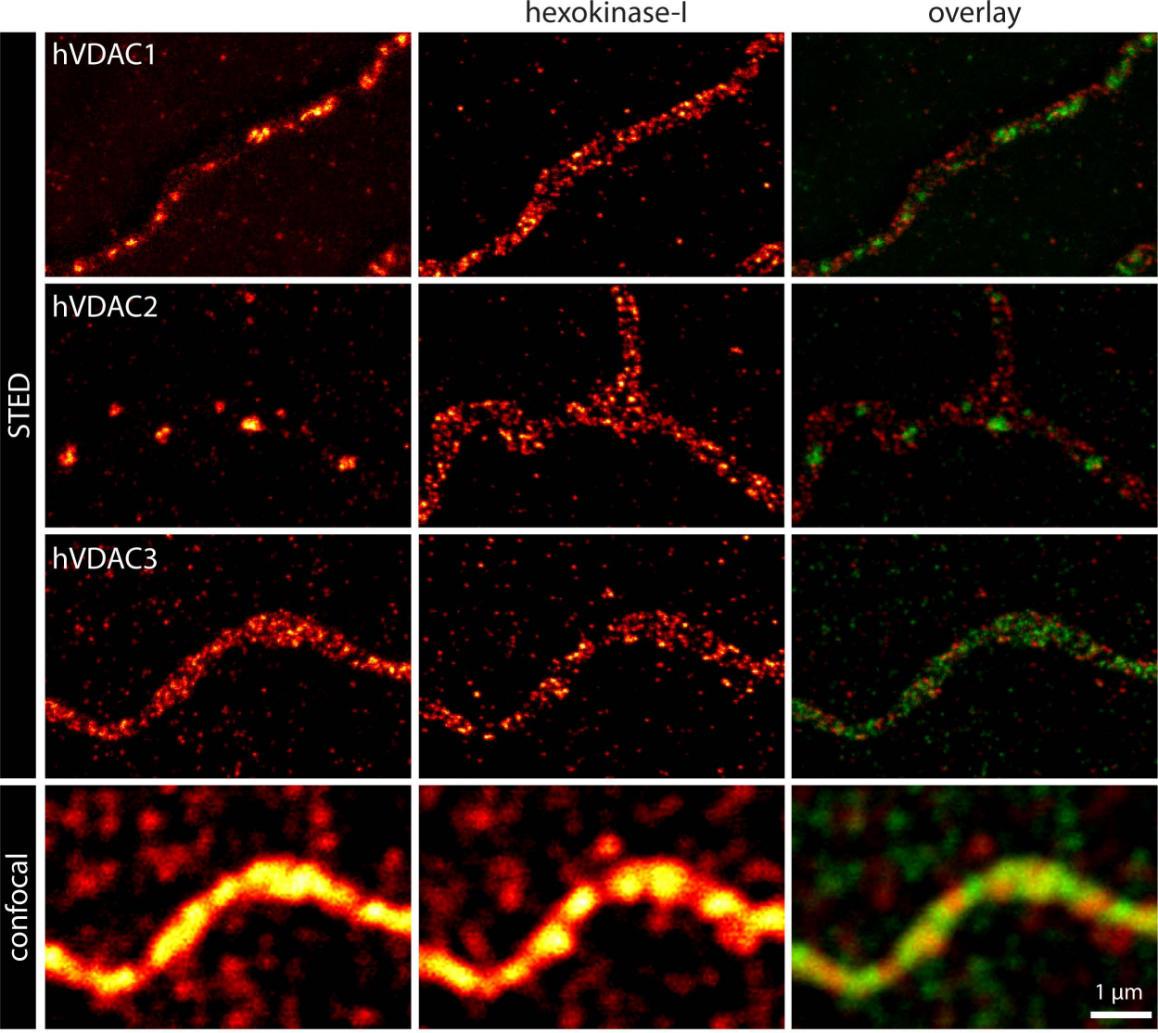
**Two-color STED microscopy of the three hVDAC isoforms and hexokinase-I reveals individual protein clusters**. U2OS cells expressing one of the three Flag-tagged hVDAC isoforms were labeled with antibodies directed against the Flag-tag and hexokinase-I. With confocal microscopy, neither individual hVDAC3 nor individual hexokinase-I clusters could be resolved (bottom row). In contrast, the corresponding STED images clearly resolved hVDAC3 and hexokinase-I clusters (third row). Two-color STED images of hVDAC1 or hVDAC2, imaged together with hexokinase-I, further shows that the hVDAC1/hVDAC2 domains are frequently composed out of smaller clusters. In the overlay the green color corresponds to the hVDAC signal and the red color to the hexokinase-I signal.

In order to analyze the distributions of the three hVDAC isoforms and that of hexokinase-I as well as their level of colocalization, we utilized a custom-built two-color STED-microscope. As fluorophores we used the red fluorescent ATTO 590 (*λ*_em _= 624 nm) and the far red fluorescent KK 114 (*λ*_em _= 662 nm) [47]. With this instrument we routinely achieved a lateral resolution in both color channels of approx. 40 nm; the axial resolution is at the confocal level (~750 nm). Typical image acquisition times were in the order of 10 min. All image data shown in this study and used for subsequent analysis are raw data. No deconvolution algorithms were utilized. Using STED microscopy we were able to resolve both individual hexokinase-I as well as hVDAC3 clusters, which were not discernible in the confocal counterpart (Fig. 5). Cluster sizes of hexokinase-I and hVDAC3 ranged generally from 90 nm down to 40 nm. The latter corresponded to the optical resolution of the microscope and should therefore be regarded as an upper bound of the actual size of these clusters. Furthermore, the STED images revealed that the large hVDAC1 or hVDAC2 domains as imaged by the confocal microscope, were frequently composed of several smaller clusters (Fig. 5). We note that overexpression of any of the hVDAC isoforms did not noticeably modify the sub-mitochondrial distribution of hexokinase-I.

Hence STED microscopy allows the visualization of individual hVDAC and hexokinase-I clusters within single mitochondria, which are not discernible with confocal microscopy.

### 3.4 The three hVDAC isoforms exhibit different degrees of colocalization with hexokinase-I

In order to determine the degree of colocalization between the three hVDAC isoforms and hexokinase-I, we analyzed two-color STED images (like those exemplified in Fig. 5) using Pearson's correlation coefficient as well as the overlap and colocalization coefficients introduced by *Manders et al. *[39]. The calculation of the parameters was restricted to the regions of the images containing mitochondria which were extracted using automated image segmentation.

**Figure 6:**
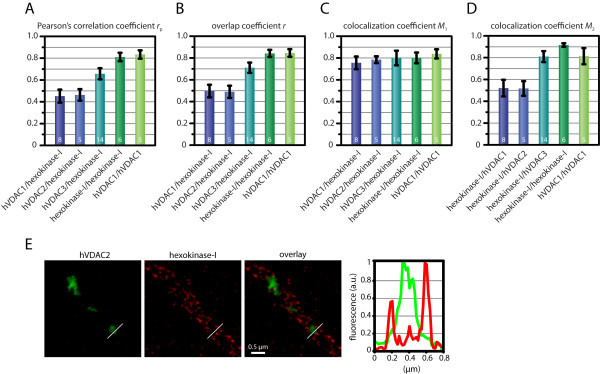
**The hVDAC isoforms exhibit different degrees of colocalization with hexokinase-I**. Quantitative colocalization analysis of any of the three hVDAC isoforms with hexokinase-I based on two-color STED images. Shown are the Pearson's correlation coefficients *r*_p _(A), the overlap coefficient *r *(B) as well as the colocalization coefficients *M*_1 _and *M*_2 _(C, D). As positive controls for full colocalization, we analyzed cells doubly labeled for hexokinase-I as well as cells co-expressing hVDAC1-Flag and hVDAC1-V5. Error bars: Standard deviation. The numbers on the columns represent the number of analyzed cells. The detailed values for all columns are given in Additional file 4. *M*_1 _and *M*_2 _may overestimate the level of colocalization because of the required thresholding. To demonstrate this effect, an intensity profile through a two-color STED image, where the cell was labeled for hVDAC2 and hexokinase-I, is shown (E). The fluorescence intensity was determined at the indicated line in the images. For details see main text.

To verify the robustness of the chosen approach, we first analyzed two control labeling experiments: In the first experiment we co-expressed two different hVDAC1 fusion constructs. Namely, we expressed hVDAC1-Flag together with hVDAC1-V5 and decorated the cells with antisera against the Flag and the V5 epitopes. The primary antibodies were detected with secondary antibodies labeled with KK 114 and ATTO 590, respectively. For the second control experiment, we did not express any fusion protein but rather decorated the cells with antibodies directed against the endogenously expressed hexokinase-I, in combination with KK 114 labeled secondary antibodies. Further, we used tertiary antibodies labeled with ATTO 590, which were directed against the secondary antibody. Assuming perfect staining and noise-free imaging, one would expect a full overlap of both colors in each control experiments We found that both Pearson's correlation coefficient and the overlap coefficient have values of ~0.83 in the control experiments (Fig. 6A, B; Additional file 4). We attribute the deviation from unity to an incomplete labeling which may be a purely statistical effect but may also be due to one antibody (which is a relatively bulky molecule of ~150 kDa) perturbing the binding of the other. Hence we conclude that with our labeling strategy, correlation/overlap coefficients of ~0.83 are the highest we can achieve with STED microscopy at a resolution of ~40 nm. Hence these values reflect quantitative colocalization.

The quantitative analysis of colocalization between the three hVDAC isoforms and hexokinase-I in terms of Pearson's correlation coefficient *r*_p _and the overlap coefficient *r *gives very similar results. For both hVDAC1/hexokinase-I and hVDAC2/hexokinase-I, we find *r*_p _≈ 0.45 and *r *≈ 0.49 (Fig. 6A, B; Additional file 4). In contrast, the degree of colocalization between hVDAC3 and hexokinase-I is significantly higher with values ranging between 0.66 (Pearson's correlation coefficient) and 0.71 (overlap coefficient). Yet, the correlation remains below that of the (positive) controls. The fact that both coefficients give the same trends further validates the analysis. Taken together, hVDAC1 and hVDAC2 have a similar partial degree of colocalization with hexokinase-I. Furthermore, there is a significantly higher, although not complete spatial overlap between hVDAC3 and hexokinase-I.

Pearson's correlation coefficient and the overlap coefficient represent the overall overlap of both analyzed signals. They are robust measures in the sense that they do not require additional parameters (e.g. thresholds) to be specified. However, they can only characterize the overall fraction of overlapping features but do not reflect differing degrees of colocalization between the two channels which may arise if, for example, the two labels have different frequencies of occurrence. Therefore, we also calculated the colocalization coefficients *M*_1 _and *M*_2 _[39] which have been tailored to reflect such different degrees of colocalization. *M*_1 _and *M*_2 _represent, respectively, the fraction of objects in channel 1 which colocalize with objects in channel 2, and vice versa. While these coefficients have a very intuitive interpretation, their calculation involves the decision at what level of fluroescence an object is considered to be present in a channel. In order to obtain unbiased thresholds, we calculated the intensity histograms of the signals (from the regions containing the mitochondria) and of the background and set the threshold to the intersection of both histograms. Using this approach, the *M*_1 _coefficients for hVDAC1, hVDAC2 and hVDAC3 with hexokinase-I ranged between 0.76 and 0.80 which is close to the positive controls (0.80-0.84, see Fig. 6C and Additional file 4). An equally high *M*_2 _coefficient was also found for hexokinase-I/hVDAC3, whereas for hexokinase-I/hVDAC1 and hexokinase-I/hVDAC2, *M*_2 _was significantly lower (0.52) (Fig. 6D). Thus, a larger fraction of hexokinase-I is colocalized with hVDAC3 than is with hVDAC1 or hVDAC2. This finding corroborates the trend already indicated by Pearson's correlation coefficient and the overlap coefficient. The consistently high *M*_1 _values, however, seem partly questionable when cross-checked by visual inspection (Figs. 5 and 6E). Visually, one may conclude that clusters of hVDAC are frequently neighbored by clusters of hexokinase-I rather than exhibiting complete colocalization. However, the exemplary intensity profile shown in Fig. 6E shows that while being weak, the level of fluorescence in the hexokinase-I channel is indeed well above the background level. Because the calculation of the colocalization coefficients only makes a binary distinction between object and background and does not weigh the different levels of intensity, the coefficients are overestimated when extended objects with many different levels of intensity are analyzed - which is the case for the hexokinase-I. Therefore, we conclude that the colocalization coefficients are not well suited for the colocalization analysis of the hVDAC isoforms and hexokinase-I.

Summarizing our colocalization analysis of the high-resolution STED images, we find that all three hVDAC isoforms colocalize with hexokinase-I to some degree. However, the extent of colocalization between hVDAC and hexokinase-I is isoform-specific. Whereas the occurrence of hVDAC3 and hexokinase-I is strongly correlated, both hVDAC1 and hVDAC2 colocalize with hexokinase-I to a significantly lower degree. Given the very high level of structural similarity of the three hVDAC isoforms, it will be enlightening to reveal the structural basis of these differences.

## 4. Summary and Outlook

We conclude that although the three hVDAC isoforms exhibit a very high degree of similarity in their primary structures, they have different sub-mitochondrial localizations. We found that when imaged with diffraction-limited confocal microscopy, hVDAC1 and hVDAC2 colocalize in the same relatively large domains, whereas hVDAC3 is quite evenly distributed on the mitochondrial surface.

Using two-color STED microscopy with ~40 nm resolution, we demonstrated different degrees of colocalization between the three hVDAC isoforms and hexokinase-I. Within this resolution range, hVDAC1 and hVDAC2 show similar degrees of colocalization with hexokinase-I. In contrast, hVDAC3 shows a substantial higher level of colocalization with hexokinase-I. This finding differs from previous biochemical data which suggested that only hVDAC1 binds hexokinase-I, but not hVDAC2 [23], and rather supports the notion that also hVDAC2 binds hexokinase-I [24]. In addition, our data show that a substantial fraction of the hexokinase-I clusters appear not to colocalize with any of the hVDAC isoforms, suggesting the existence of distinct hexokinase-I pools within mitochondria.

This study exemplifies that quantitative colocalization analysis of high resolution STED image data is a powerful tool to study the complex spatial relationship of proteins in intact cells. The image data cannot render biochemical interaction maps superfluous, but they represent an additional level of information. Notably, high-resolution images can provide novel insights on sub-cellular or sub-organellar heterogeneities in the spatial interaction of proteins. Such information is difficult or even impossible to obtain by biochemical analysis and is frequently concealed by the limited spatial resolution when using conventional microscopy.

## Competing interests

The authors declare that they have no competing interests.

## Supplementary Material

Additional file 1 **Heterogeneity of the hVDAC3 distribution induced by co-expression with hVDAC1**. Confocal microscopy of U2OS cells transfected with hVDAC3-Flag alone (left image) or with hVDAC3-Flag together with hVDAC1-V5 (right image). For visualisation of hVDAC3, cells were decorated with antisera against the Flag tag. Comparing both images reveals that some heterogeneity of the hVDAC3 distribution is induced by the co-expression of hVDAC1-V5. Click here for file

Additional file 2 **Crosstalk between the green and the red channel in the confocal microscope**. In order to determine the level of crosstalk between both detection channels, cells were labeled as for two-color confocal imaging (see Fig. 3) with the exception that we used only one primary antibody and the corresponding secondary antibody. All imaging parameters were the same as for two-color confocal imaging. No detectable crosstalk was observed. Click here for file

Additional file 3 **Colocalization analysis of the three hVDAC isoforms based on confocal images**. (A) Quantitative colocalization analysis of hVDAC1 with hVDAC2 and hVDAC3. Shown are the Pearson's correlation coefficient *r*_p_, the overlap coefficient *r *as well as the colocalization coefficients *M*_1 _and *M*_2_. As positive control for full colocalization, we analyzed cells co-expressing hVDAC1-Flag and hVDAC1-V5. Error bars: Standard deviation. For each column 10 cells were analyzed. (B) Detailed values graphically represented in (A). Click here for file

Additional file 4 **Colocalization analysis based on two-color STED images**. Detailed values as graphically represented in Fig. 6A-D in the main text. For details see caption to Fig. 6. Click here for file
