# Supplementary figures and images for: Two-color STED microscopy reveals different degrees of colocalization between hexokinase-I and the three human VDAC isoforms
Source: PMC Biophys. 2010 Mar 5;3:4. doi: 10.1186/1757-5036-3-4 (PMC2838807; doi:10.1186/1757-5036-3-4)

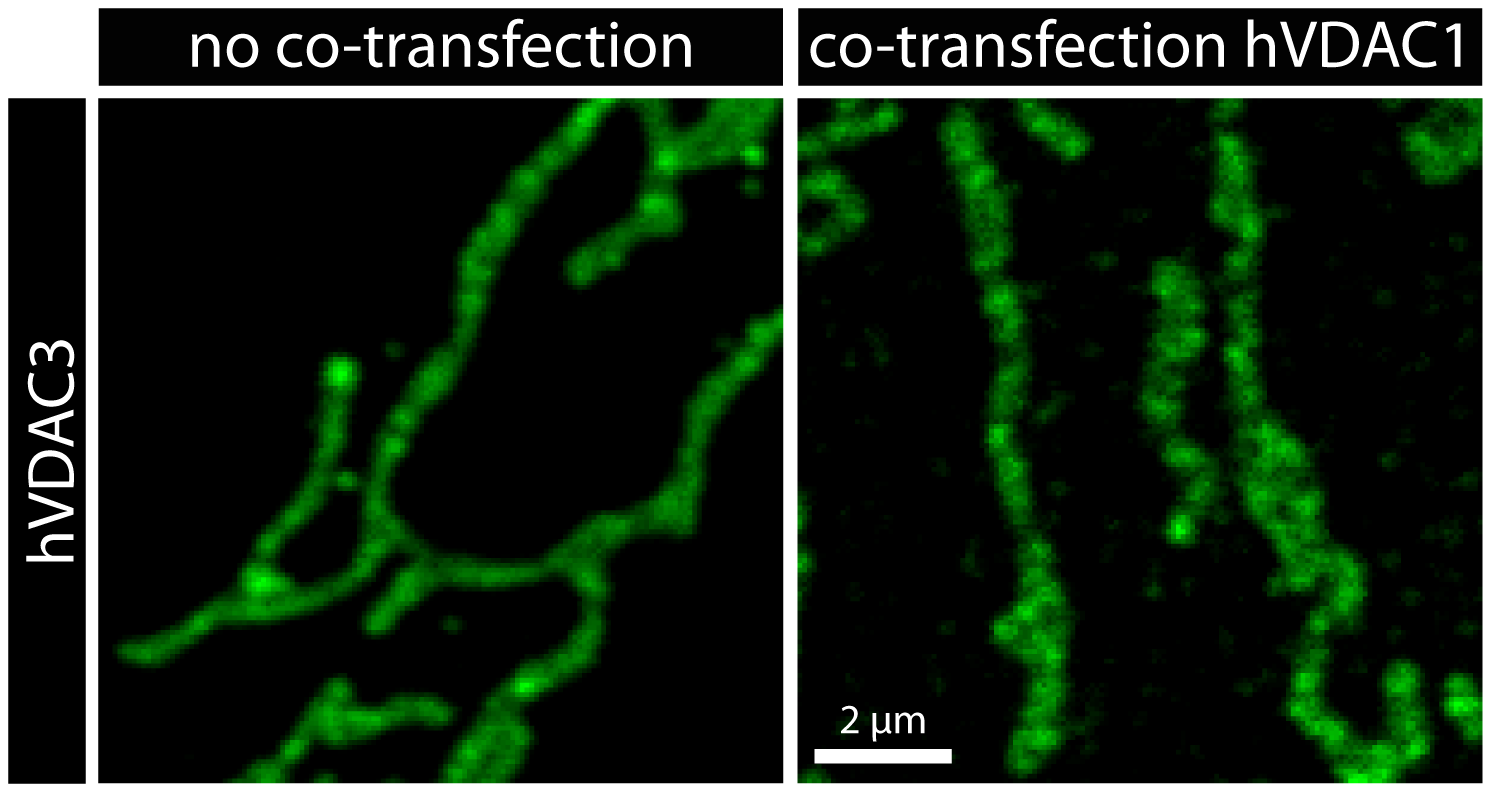

Supplement: Additional file 1 — Heterogeneity of the hVDAC3 distribution induced by co-expression with hVDAC1. Confocal microscopy of U2OS cells transfected with hVDAC3-Flag alone (left image) or with hVDAC3-Flag together with hVDAC1-V5 (right image). For visualisation of hVDAC3, cells were decorated with antisera against the Flag tag. Comparing both images reveals that some heterogeneity of the hVDAC3 distribution is induced by the co-expression of hVDAC1-V5. [file 1757-5036-3-4-S1.TIFF]

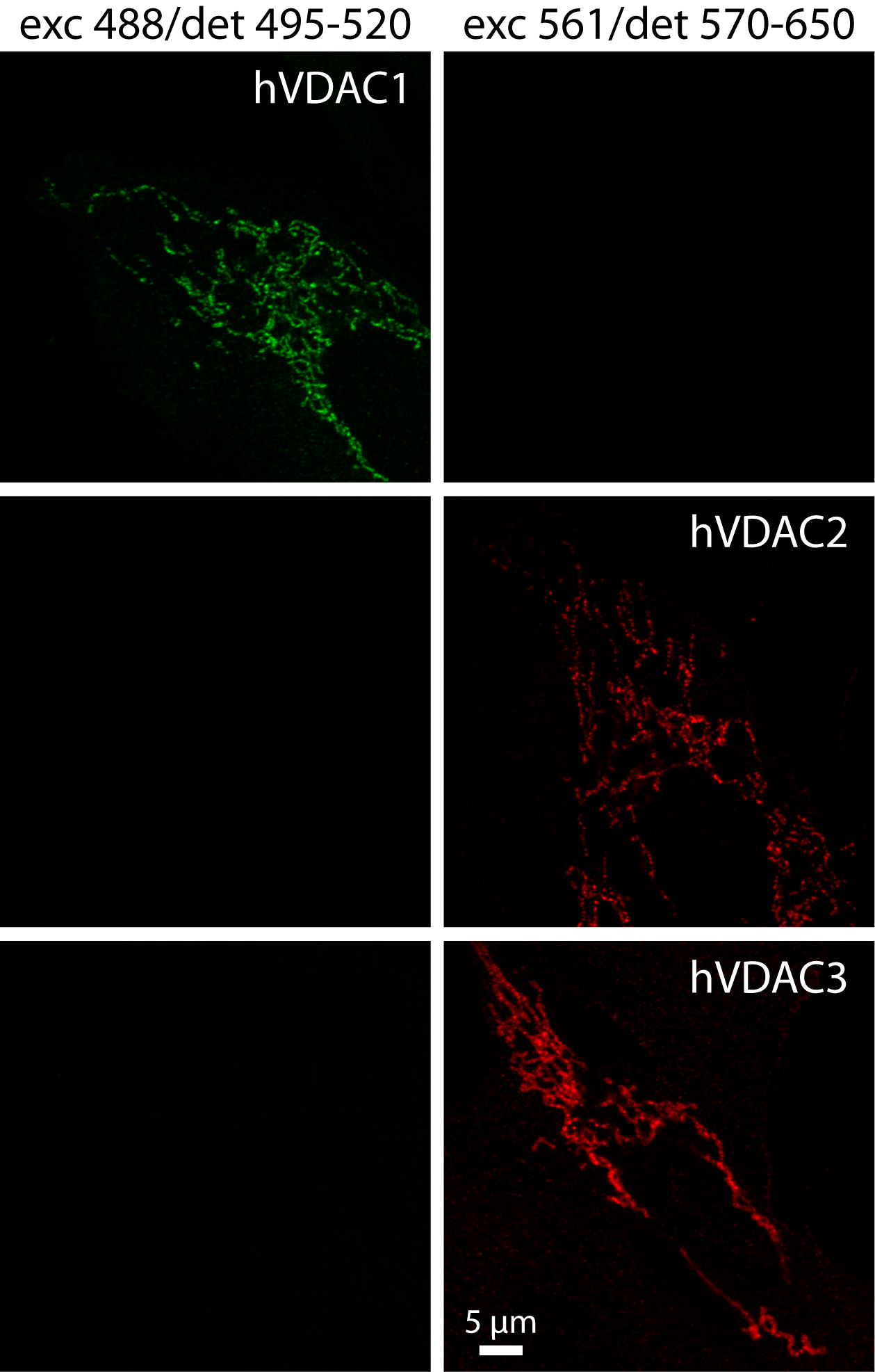

Supplement: Additional file 2 — Crosstalk between the green and the red channel in the confocal microscope. In order to determine the level of crosstalk between both detection channels, cells were labeled as for two-color confocal imaging (see Fig. 3) with the exception that we used only one primary antibody and the corresponding secondary antibody. All imaging parameters were the same as for two-color confocal imaging. No detectable crosstalk was observed. [file 1757-5036-3-4-S2.TIFF]

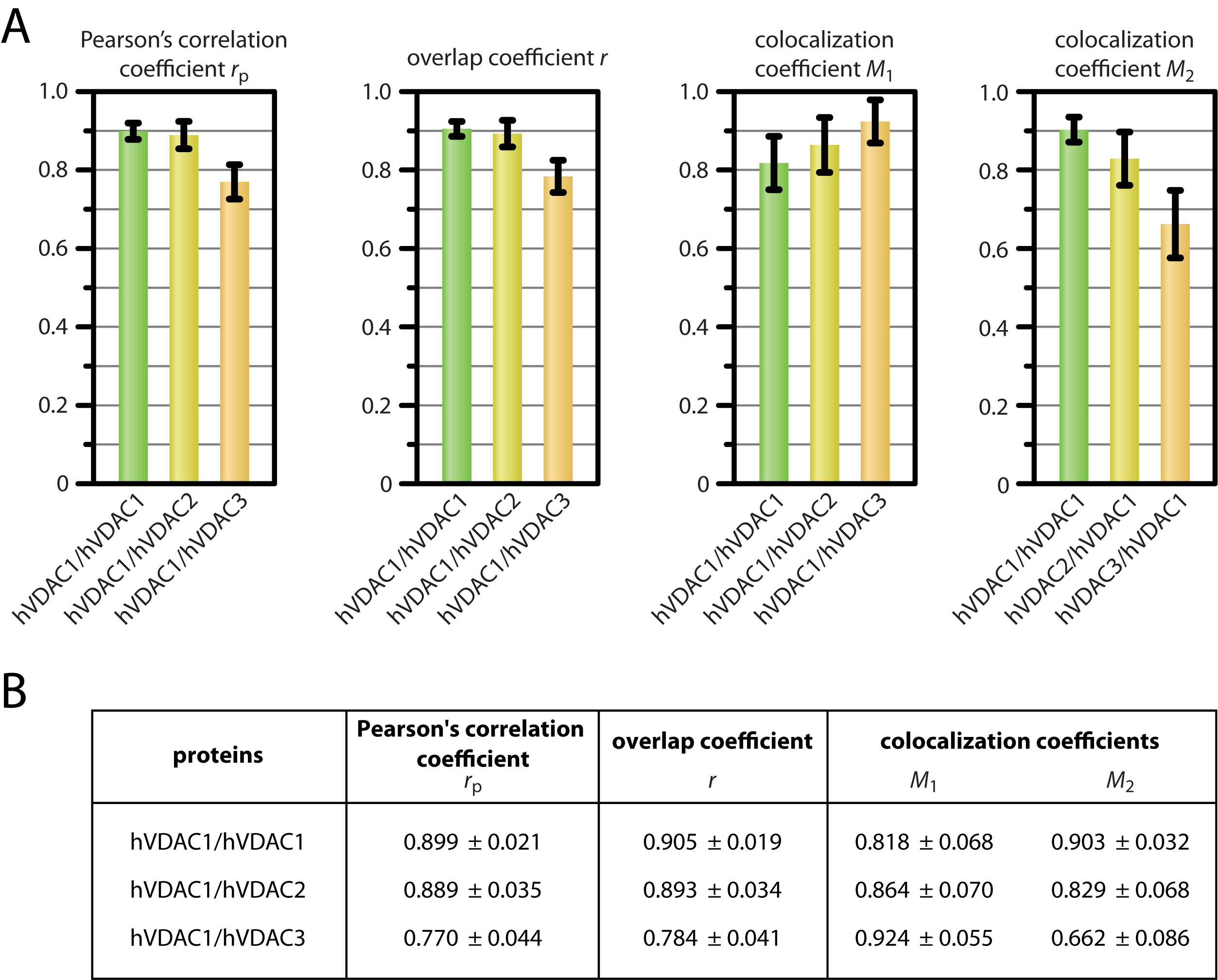

Supplement: Additional file 3 — Colocalization analysis of the three hVDAC isoforms based on confocal images. (A) Quantitative colocalization analysis of hVDAC1 with hVDAC2 and hVDAC3. Shown are the Pearson's correlation coefficient rp, the overlap coefficient r as well as the colocalization coefficients M1 and M2. As positive control for full colocalization, we analyzed cells co-expressing hVDAC1-Flag and hVDAC1-V5. Error bars: Standard deviation. For each column 10 cells were analyzed. (B) Detailed values graphically represented in (A). [file 1757-5036-3-4-S3.TIFF]

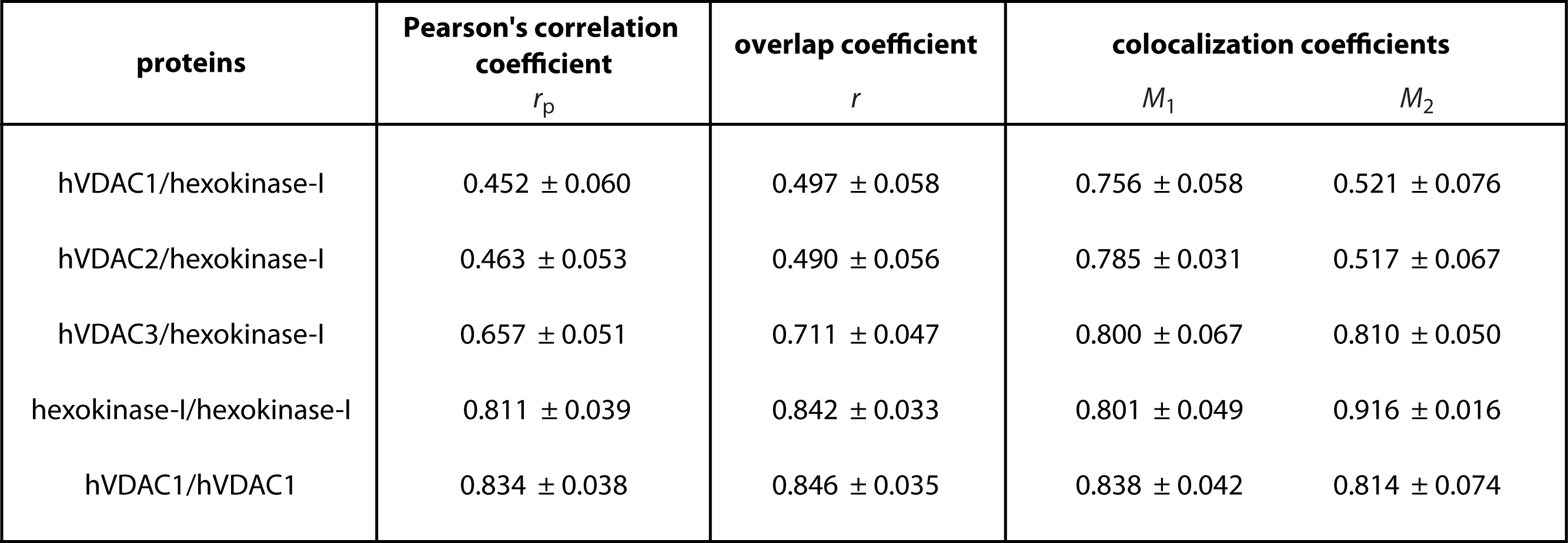

Supplement: Additional file 4 — Colocalization analysis based on two-color STED images. Detailed values as graphically represented in Fig. 6A-D in the main text. For details see caption to Fig. 6. [file 1757-5036-3-4-S4.TIFF]
